# The Bandim TBscore – reliability, further development, and evaluation of potential uses

**DOI:** 10.3402/gha.v7.24303

**Published:** 2014-05-22

**Authors:** Frauke Rudolf

**Affiliations:** 1 Bandim Health Project, INDEPTH Network, Bissau, Guinea-Bissau; 2 Department of Infectious Diseases, Aarhus University Hospital, Aarhus, Denmark

**Keywords:** clinical score, reliability, low-resource setting, triage, mortality prediction, health status indicator, diagnosis, case finding

## Abstract

**Background:**

The tuberculosis (TB) case detection rate has stagnated at 60% due to disorganized case finding and insensitivity of sputum smear microscopy. Of the identified TB cases, 4% die while being treated, monitored with tools that insufficiently predict failure/mortality.

**Objective:**

To explore the TBscore, a recently proposed clinical severity measure for pulmonary TB (PTB) patients, and to refine, validate, and investigate its place in case finding.

**Design:**

The TBscore’s inter-observer agreement was assessed and compared to the Karnofsky Performance Score (KPS) (paper I). The TBscore’s variables underlying constructs were assessed, sorting out unrelated items, proposing a more easily assessable TBscoreII, which was validated internally and externally (paper II). Finally, TBscore and TBscoreII’s place in PTB-screening was examined in paper III.

**Results:**

The inter-observer variability when grading PTB patients into severity classes was moderate for both TBscore (*κ*_W_=0.52, 95% CI 0.46–0.56) and KPS (*κ*_W_=0.49, 95% CI 0.33–0.65). KPS was influenced by HIV status, whereas TBscore was unaffected by it. In paper II, proposed TBscoreII was validated internally, in Guinea-Bissau, and externally, in Ethiopia. In both settings, a failure to bring down the score by ≥25% from baseline to 2 months of treatment predicted subsequent failure (*p*=0.007). Finally, in paper III, TBscore and TBscoreII were assessed in health-care-seeking adults and found to be higher in PTB-diagnosed patients, 4.9 (95% CI 4.6–5.2) and 3.9 (95% CI 3.8–4.0), respectively, versus patients not diagnosed with PTB, 3.0 (95% CI 2.7–3.2) and 2.4 (95% CI 2.3–2.5), respectively. Had we referred only patients with cough >2 weeks to sputum smear, we would have missed 32.1% of the smear confirmed cases in our cohort. A TBscoreII>=2 missed 8.6%.

**Conclusions:**

TBscore and TBscoreII are useful monitoring tools for PTB patients on treatment, as they could fill the void which currently exists in risk grading of patients. They may also have a role in PTB screening; however, this requires our findings to be repeated elsewhere.

Tuberculosis (TB) is an ancient disease that has plagued mankind through its existence (1). Despite a cure being developed in the 1950s, TB still ranks number 10 on the list of ‘global death ranks for the top 25 causes’ (2), and in 2012, nearly 8.6 million people developed TB, whereas 1.3 million died from the disease (3). The target of halving TB prevalence by 2015 will not be reached (3).

Low detection rates and therefore stable sources for infection, the HIV/AIDS pandemic, low cure rates, and disorganized and insufficiently resourced TB control programs (4) maintain the strength of the epidemic. Increasing resistance to currently available anti-TB drugs (5) and insensitivity of the only widely available diagnostic tool, sputum smear microscopy (6), have revived research. The current research focus is mainly directed toward development of new drugs and vaccines although there have been calls for better diagnostic tools (4, 6–11) and repeated propositions to use existing algorithms and tools to improve case management and detection (12, 13). A patient diagnosed with pulmonary TB (PTB) is treated with antibiotics for 6 months. An estimated 4% die while on treatment (14) – deaths that could have been avoided if high-risk cases were to be identified early (15).

The aim of this study was to evaluate, refine, and explore possible applications of the TBscore, a previously proposed clinical score (16) used to assess mortality and treatment failure risk for TB patients on treatment.

## Background

### Clinical prediction rules in general and in TB

Clinical prediction rules (CPRs) use clinical findings to diagnose a disease or predict an outcome (17). They are useful when clinicians fail to identify relevant but under-diagnosed conditions (18) and clinical decision making is complex (19). Further, they may guide less experienced examiners (20) through the right diagnostic pathway. A frequently used CPR in TB is the Karnofsky Performance Score (KPS) (21, 22), which has been used as an indicator for disease severity (23), as treatment response measure (24, 25), and to predict mortality (26). The KPS is a subjective rating tool consisting of performance from 0 to 100% according to the ability to perform daily activities, to work, need for assistance, and presence of disease-related symptoms (22).

Table 1 shows CPRs for PTB published over the recent years; most of them were developed to aid the clinician to decide if patients admitted to hospitals in low- and medium-incidence settings should be placed in isolation (27–31). Others are used on initially sputum smear-negative (SN) patients to improve and accelerate diagnosis of PTB (32–34). Few have tried to combine signs and symptoms into a CPR to screen for PTB (35–38) and only two CPRs to monitor TB treatment response have been proposed (16, 39). Horita et al. (39) suggest a score consisting of age (in years), oxygen requirement, albumin concentration (g/dl), and activity of daily living. The TBscore proposed by Wejse et al. (16) consists of five symptoms (cough, hemoptysis, dyspnea, chest pain, and night sweats) and six signs (pale inferior conjunctivae, pulse >90 per minute, positive finding at lung auscultation, temperature >37°C [axillary], body mass index [BMI] <18/<16, and mid-upper-arm circumference [MUAC] <220 mm/<200 mm). Each variable contributes with one point while BMI and MUAC contribute with an extra point, if <16/<200 mm; hence, the maximum score 13 (Table 2). The original three severity classes (SC) were SCI, TBscore 0–5; SCII, TBscore 6–7, and SCIII, TBscore ≥8 (16).

**Table 1:** Existing CPRs for TB

Authors, year	Aim	Type of patient	Outcome predicted
Aguiar et al. (27)	Predictive model for PTB for a more rational decision on the use of isolation rooms.	Patients suspected of having TB admitted to isolation rooms (Cough >2 weeks+radiologic abnormality, or respiratory symptom+confirmed/suspected HIV-infection).	PTB (Isolation of *Mtb* in solid culture; findings of granulomatous inflammation with caseous necrosis in respiratory tissue biopsy; improvement of respiratory symptoms within 60 days of TB treatment.
Rakoczy et al. (28)	Stratifying patients suspected of having TB (use of isolation rooms).	All adult in-patients with newly diagnosed PTB on the basis of positive results from sputum or bronchoscopes fluid culture (retrospective).	AFB positive results from sputum or bronchoscopy fluid culture.
Solari et al. (29)	Guides decision to isolate patients with pulmonary complaints suggestive of PTB.	Aged 18 years+productive cough for ≥7 days; cough of any duration+constitutional symptoms (fever ≥3 days, night sweats, or weight loss ≥3 kg in the previous month); hemoptysis or a differential diagnosis of PTB from the attending physician.	Culture positive TB.
Wisnivesky et al. (31)	Identify early predictors of the need for respiratory isolation.	Patients with positive culture compared to patients who had handed in a sputum sample but were culture negative.	Culture positive PTB.
Alavi-Naini et al. (32)	Identify characteristics that identify individuals with SN PTB.	Negative sputum smears for AFB and remaining symptomatic after a course of broad spectrum antibiotics.	Culture confirmed SN PTB.
Mello et al. (33)	Predictive model for SN PTB among outpatients.	Patients with cough ≥3 weeks, and two consecutive samples of spontaneous sputum that were AFB-negative, or an absence of expectorated sputum.	Confirmed SN PTB: positive culture for *Mtb* in respiratory specimen; Presumptive SN PTB: clinical improvement after 3 months of anti-TB treatment.
Soto et al. (34)	CPR to diagnose SN PTB.	Patients admitted to emergency department with cough ≥1 week+one or more of the following: fever (38°C), weight loss (≥4 kg in a month), constitutional symptoms (malaise or hyporexia for ≥2 weeks) or dyspnea.	Culture confirmed SN PTB.
Alcântara et al. (35)	Identify socio-demographic, clinical, and behavioral factors that are associated with PTB.	Older than 14 years of age; having sought medical attention spontaneously; presenting with cough ≥2 weeks; willing to provide at least one sputum sample and undergo chest X-ray.	Positive culture for *Mtb* or an AFB-positive sputum smear; Clinical and radiological characteristics suggestive of PTB+improvement after 6 months of anti-TB treatment alone.
Fournet et al. (36)	Identify a subgroup of ‘TB suspects’ who qualify for diagnostic investigations.	Male in-mates aged ≥18 years.	Active TB: sputum smear – culture positive. Or a positive clinical and radiological response to specific treatment.
Corbett et al. (37)	Investigate the effect of HIV on the prevalence of TB symptoms and on the diagnostic utility of different screening strategies.	Individuals with current cough, hemoptysis during the previous year, self-reported fever or ‘hot body’, night sweats and a subjective report of weight loss, or who tested positive on sputum culture during screening (i.e. culture-positive).	Sputum culture – positive for *Mtb* on two or more occasions; Failure to respond to broad-spectrum antibiotics+response to TB treatment at 1 month; Culture-negative for *Mtb+*radiological evidence of TB or the presence of TB-symptoms, failure to respond to antibiotics+response to TB treatment at 1 month.
Hanifa et al. (38)	Determine prevalence of previously undiagnosed active TB among ART-eligible adults. Evaluate the performance of combinations of symptoms and standard investigations in the diagnosis of TB.	ART-eligible (WHO Stage 4 or CD4<200 cells/mm^3^) adults (aged >17 years).	Compatible clinical or radiological features and sputum culture-positive for *Mtb* (definite PTB); Sputum smear-positive, culture-negative (probable PTB); no other cause of disease found+clinical improvement after 2 months of anti-TB treatment, or lost to follow-up or died before 2 months (possible PTB).
Horita et al. (39)	Estimating the prognosis of an individual TB patient.	Smear positive, HIV uninfected, treatment susceptible.	Death
Wejse et al. (16)	Develop a simple clinical score for repeated clinical status – evaluation of TB patients during treatment.	Pulmonary TB patients (smear positive and negative), HIV-infected and -uninfected.	Change during TB treatment. Mortality.

TB=tuberculosis; PTB=pulmonary TB; CPR=clinical prediction rule; SN=smear negative; *Mtb*=mycobacterium tuberculosis; AFB=acid fast bacilli; ART=anti retroviral treatment; HIV=human immunodeficiency virus.

**Table 2:** TBscore and TBscoreII.

	Score	
	
Variables included	TBscore	TBscoreII
*Symptoms*		
Cough	1	1
Hemoptysis	1	–
Dyspnea	1	1
Chest pain	1	1
Night sweats	1	–
*Signs*		
Anemia	1	1
Pulse >90 beats/min	1	–
Positive finding at lung auscultation	1	–
Temperature >37°C	1	–
BMI<18	1	1
BMI<16	1	1
MUAC<220 mm	1	1
MUAC<220 mm	1	1
Total number of points possible	13	8

BMI=body mass index; MUAC=mid upper arm circumference.

### Areas of use for the TBscore – stagnated case detection rates and deaths during treatment

The newest estimates by the WHO state that one third of all active TB cases are not properly diagnosed and hence not detected (3). Gold standard for TB diagnosis is sputum culture. However, most settings are still relying on sputum smear microscopy (3), a 125-year-old method which misses half of the cases (6) and even more if the demand for sputum smears exceed laboratory capacity (40). Not finding *Mtb* in a sputum smear does not exclude TB as the possible diagnosis (41); in HIV-infected individuals, the bacteria are often not found in a sputum smear (42, 43). If SN, the patient is prescribed antibiotics and/or referred to chest x-ray (CXR), which is unspecific and hard to interpret for inexperienced observers, especially if the patient is HIV co-infected (41, 44). A recently published review on TB diagnostics states that ‘Simply increasing case detection rates through existing diagnostics will go a long way in reducing transmission of PTB’ (12). This, however, requires an increased awareness toward PTB symptoms at health-care facilities and systematic screening routines.

While on treatment, an estimated 4% of TB patients die due to the disease, 3% of the HIV-uninfected and 9% of the HIV-infected patients (14). A previous review found case fatality rates (CFR) of 1.8–33% (15). The review emphasizes that there is a need to improve recognition of TB patients at the risk of dying while being treated, stating that ‘in low-resource settings with strained infrastructure, development of a simple clinical tool to streamline prioritization of intensified follow-up of high-risk patients would be of great benefit’ (15).

The current method to evaluate effect of treatment in PTB patients is repeated sputum smear examinations at second, fifth, and sixth month of treatment for initially sputum smear-positive patients (45). This approach has been shown to be insensitive (46–49); finding bacteria in a sputum smear does not mean that the bacteria found are viable (50). Also, smear conversion is influenced by age and height of bacillary load at treatment initiation (50). Since SN patients are excluded in this recommendation, the WHO suggests weight gain as prognostic marker for this group of patients (45). Weight has been shown to be insufficient in predicting overall outcome (51), since the patients mostly gain fat mass (52) masking an eventual loss of muscle and possibly also organ tissue (53). Further, there is no clear definition on how to use weight gain as a prognostic marker (i.e. how much gain is enough).

## Aim

The overall aim of the PhD project was to refine and explore the TBscore to define areas of use.

The specific aims were (Table 3):

- To assess inter-observer variation for the TBscore used by physicians with different backgrounds and compare the TBscore to another disease severity rating tool (i.e. the KPS) (54).
- To further develop and refine the TBscore to improve inter-observer variation and validate the proposed TBscoreII internally and externally (55).
- To investigate the performance of TBscore and TBscoreII and compare them to other PTB screening tools (56).

**Table 3:** Overview of the studies constituting the thesis

Study	Research question	Methodology	Results
I: Bandim TB score: reliability and comparison with the KPS (54).	Assessing the variability between two physicians in performing the Bandim TB score (TBscore). To compare the TBscore to the KPS.	Prospective assessment of the TBscore and the KPS of 100 PTB patients at inclusion and/or at follow-up visits (at second, fourth, and sixth month of treatment).	Inter-observer variability of the TBscore varied from slight to almost perfect. The grading into severity classes showed moderate agreement for TBscore and KPS. The ICC was larger for the TBscore than for the KPS.
II: TBscore II: refining and validating a simple clinical score for treatment monitoring of patients with PTB (55).	Simplify the TBscore, proposing TBscoreII. Validate TBscoreII internally and externally.	EFA on observational data from 565 PTB patients from Bissau. Validation of TBscore and TBscoreII with observational data from 488 Guinean and 432 Ethiopian PTB patients.	Proposal of TBscoreII. Moderate Cohen’s ES for TBscore-change in Bissau and large ES in Gondar between baseline and second month of follow-up. Prediction of failure and mortality by TBscore and TBscoreII, significant in Bissau.
III: Can TB case-finding among health-care-seeking adults be improved? – Observations from Bissau (56).	Assess the potential benefit of elsewhere applied predictors and TBscore/TBscoreII, in PTB case finding.	Observational prospective cohort study assessing TBscore/TBscoreII and other clinical predictors in adults seeking health care for cough, weight loss and/or sputum production (PTB suspects).	Cough> 2 weeks had the best diagnostic ability overall. TBscore<3 excluded PTB. A TBscoreII ≥3 had largest diagnostic ability in HIV-infected patients. Absence of self-reported weight loss excluded PTB best in HIV-infected patients. Applying cough >2 weeks as smear microscopy trigger missed 32.1% while applying a TBscoreII ≥2 missed 8.6% of the smear positive PTB cases.

TB=tuberculosis; PTB= pulmonary TB; KPS=Karnofsky Performance Score; ICC=intra-class correlation coefficient; ES=effect size; EFA=exploratory factor analysis; HIV=human immunodeficiency virus.

## Material and methods

### Setting and study population

The studies took place at the Bandim Health Project (BHP) in Bissau, Guinea-Bissau, with an estimated TB incidence rate of 238/100,000 population and a case detection rate of 56% in 2011 (57).

The BHP is a health and demographic surveillance site (HDSS) and part of INDEPTH (International Network for the Demographic Evaluation of Populations and Their Health in Developing Countries). It has registered around 100,000 people in six suburbs of the capital Bissau since 1978. In 1996, a TB surveillance program was implemented, registering TB patients living and starting treatment in the BHP area. Since 2010, adult patients (≥15 years) from the area seeking health care at health centers and confirming to cough, weight loss, or expectoration of sputum are included in the PTB suspects (PTBS) cohort.

### Data and applied routines

All patients in the TB cohort in Bissau are followed throughout their treatment, with clinical controls every second month.

Data for the study on inter-observer variation were collected by scoring all patients coming for inclusion or follow-up visit in separate rooms at the same health center, within 30 min.

Revision of the TBscore was based on data from both inpatients and outpatients in Bissau and from adult TB patients (≥18 years) attending the Directly Observed Treatment Short-course (DOTS) clinic at Gondar University Hospital, Ethiopia. In Ethiopia, the incidence rate of TB was estimated to be 258/100,000 population and the case detection rate was 72% in 2011 (58).

To explore the TBscores’ place in case finding, we collected clinical data from 1,089 PTBS, referring all consenting PTBS to sputum smear microscopy and HIV-testing and carrying out a follow-up visit 2 weeks after the first encounter. If symptoms persisted (i.e. hemoptysis, persistent cough, or two or more than two of the following symptoms: chest pain, dyspnea, night sweats, fever and/or weight loss), the patient was treated with amoxicillin (1.5 g/day, for 7 days) and referred to CXR. After finishing the amoxicillin treatment, another consultation was carried out by an experienced TB physician who decided further action; for example, final diagnosis or a second treatment with erythromycin (1.5 g/day, for 7 days) followed by another CXR and a final diagnosis.

### HIV diagnosis

Consenting PTB patients and PTBS from Bissau were HIV-tested using Determine™ HIV-1/2 (Alere Inc., MA, USA) and positive results were confirmed with SD Bioline HIV 1/2 3.0 (Standard Diagnostics Inc., Korea).

Ethiopian patients were HIV tested as part of provider-initiated HIV counseling and testing program (PIHCT) using Determine (HIV-1/2 Ag/Ab Combo, FL, USA), Capillus (Trinity Biotech USA Inc, NY, USA), and Unigold (Trinity Biotech USA INC, NY, USA).

### Data analysis

All analyses was carried out in Stata Statistical Software version 11 and 12 (Stata Corporation, TX, USA). All values are displayed with 95% Confidence Intervals (95% CI), when applicable. A two-tailed p≤0.05 was considered significant.

Inter-observer variation was determined using the kappa statistic with linear weights, penalizing disagreement in terms of seriousness (59, 60), and ranked according to Viera and Garret (60). To assess the ratio of variance between individuals and the total variance (between individuals and between measurements), we calculated the intra-class correlation coefficient (ICC) (61). We plotted the differences between the two observers’ scorings against their mean in a Bland–Altman plot to uncover potential systematic differences and show the overall distribution of scores (62, 63).

Refinition of the TBscore was done applying an exploratory factor analysis (EFA), clarifying the underlying structure of the variables (64), which are grouped according to their clustering pattern, under not measured underlying constructs (latent factors), with a correlation of ≥0.4 between factor and variable defined as significant (64). Responsiveness was evaluated by Cohen’s effect size (ES), that is, the difference between the mean baseline and follow-up scores divided by the standard deviation of the baseline scores and ranked according to Husted et al. (65).

To assess the relationship of the items toward PTB diagnosis, we used logistic regression analysis. The discriminating ability of significant items with regard to PTB-diagnosis was assessed with receivers operating characteristic (ROC) analysis (66). Negative predictive value, that is, the probability of a suspect in our cohort not having PTB if the item was absent, and the negative likelihood ratio (LR), that is, the ratio between the false negative tests among patients having the disease and true negative tests among healthy patients, were assessed to describe the items ability to exclude PTB.

### Ethical considerations

The studies were approved by the Ministry of Health in Guinea-Bissau/the Ethics Committees at Gondar College of Medical Sciences, Ethiopia, and the Central Ethical Committee in Denmark. Patients provided oral and written informed consent in all studies; for adolescents aged 15–17, assents from their parents or legal guardian was required. All participants were offered HIV-testing with pre- and post-test counseling.

## Results

### The Bandim TB score: reliability and comparison with the KPS (54)

The study included 100 PTB patients with a mean age of 33 years (95% CI 31–36) and an HIV infection prevalence of 28%. The analysis was done on 191 double scorings.

The weighted agreement when placing the patients in SC was moderate for both scores (TBscore: κ_w_=0.52 [95% CI 0.45–0.60]; KPS: κ_w_=0.49 [95% CI 0.33–0.65]). Agreement between the two observers was assessed for each variable being part of the TBscore. Almost perfect agreement was found for cough, MUAC<220 mm, and MUAC<200 mm while it was slight for hemoptysis.

The scorings carried out with the TBscore where distributed between all three SCs. However, the KPS scorings only yielded one observation in SCIII, placing almost all patients in SCIII.

While 63% (ICC=0.632) of the variance in KPSs were due to true variance, the variance between the observers when scoring with the TBscore was for 82%, a result of the true variance between the scored patients (ICC=0.822). The Bland–Altman analysis revealed that one observer gave 25% fewer TBscore-points than the other, whereas for the KPS one observer gave 1% less (*p*=0.82) points than the other, indicating a systematic difference between the observers when scoring with the TBscore.

When assessing the scores’ ability to predict unsuccessful outcome (i.e. treatment failure, death, default), a trend was seen for the TBscore (*p*=0.082) but not for the KPS (*p*=0.228).

### TBscoreII: refining and validating a simple clinical score to monitor PTB patients on treatment (55)

Clinical data from 1,070 Guinean and 432 Ethiopian PTB patients were used to refine the TBscore. While Ethiopian patients were younger (32 years; 95% CI 30–33) than the patients from Guinea-Bissau (36 years; 95% CI 35–36), had a higher HIV-prevalence (31% vs. 29%, *p*=0.021), higher percentage of sputum smear positivity (76% vs. 70%, *p*=0.003), a higher TBscore (7.2; 95% CI 7.0–7.5) versus (5.7; 95% CI 5.6–5.8) and a higher TBscoreII (4.6; 95% CI 4.5–4.8) versus (3.6; 95% CI 3.5–3.7), no significant difference was found regarding gender distribution (41% females vs. 38% males, *p*=0.423) and successful (completed or cured) treatment outcome (85% vs. 83%, *p*=0.362).

The underlying pattern of the TBscores variables was explored in a random sample of 565 PTB patients from Bissau. It seemed that hemoptysis, pulse, and temperature were not to be part of the construct explained by the underlying factors. Excluding the items found to have been agreed on less than substantial in the inter-observer analysis in paper I in addition to the ones not related to the underlying constructs, we proposed TBscoreII consisting of cough, dyspnea, chest pain, anemia, BMI<18, BMI<16, MUAC<220 mm, and MUAC<200 mm.

The inter-observer agreement of TBscoreII grading patients into SC was found to be substantial (κ_w_=0.72; 95% CI 0.66–0.79). The ES was moderate for TBscore and TBscoreII from baseline to 2-month follow-up in Bissau while it was large for TBscore and moderate for TBscoreII in Gondar. From baseline to end of treatment, the ES was large for TBscore and moderate for TBscoreII in both settings. Failure to decrease TBscore to ≥25% from treatment start to second month of treatment was significantly associated with subsequent treatment failure (*p*=0.007 in Bissau and Gondar). For TBscoreII, the association was significant only in Gondar (*p*<0.001). While a failure to decrease TBscore to ≥25% during the first 2 months was significantly associated (*p*=0.007) with subsequent mortality in Bissau, the association was significant only for TBscoreII in Gondar (*p*=0.008).

**Fig. 1:**
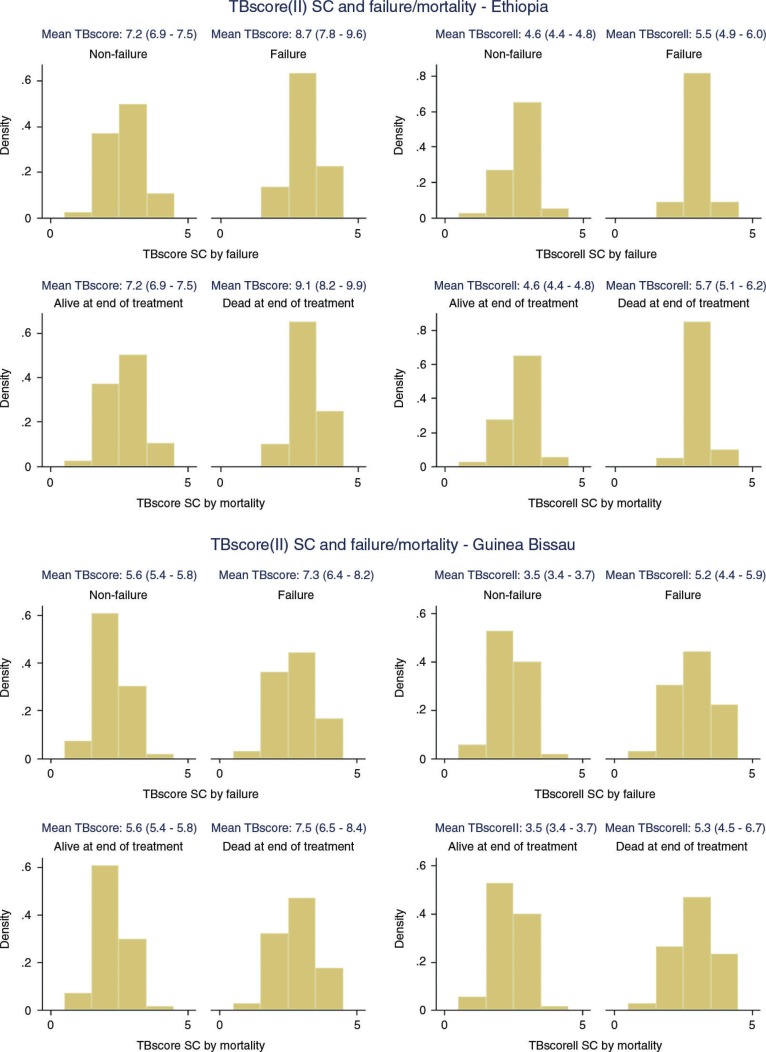
TBscore(II) at treatment start and subsequent failure/mortality.

In both settings, TBscore and TBscoreII at the beginning of treatment were significantly higher in patients failing on treatment or dying while on treatment (Fig. 1).

### Can TB case finding among health-care-seeking adults be improved? Observations from Bissau (56)

The study cohort consisted of 1,089 patients presenting with cough and/or weight loss and/or expectoration with a mean age of 34 years (95% CI 33–35 years), and a HIV-infection rate of 15.1%.

A total of 107 patients were diagnosed with PTB; 76.4% sputum smear positive and 25.2% HIV infected. At follow-up after 2 weeks from first encounter, symptoms persisted in 89 (9.7%) of the initially SN or smear result lacking PTBS. Of those, 82 (92.1%) were treated with amoxicillin and had a CXR taken before and after. Following through the algorithm, 11 were diagnosed with SN PTB, 6 were asymptomatic, 26 did not have PTB, and in 33 PTB could not be excluded at the second consultation following amoxicillin treatment. All 33 inconclusive cases were treated with erythromycin and had a third CXR taken. The final diagnosis was given at a third consultation; for 15 it was PTB.

### The role of TBscore and TBscoreII in PTB screening and diagnosis

PTBS diagnosed with PTB had a significantly higher TBscore (4.9; 95% CI 4.6–5.2) versus (3.9; 95% CI 3.8–4.0) and TBscoreII (3.0; 95% CI 2.7–3.2) versus (2.4; 95% CI 2.3–2.5) than those not diagnosed with PTB. More than one third (34.6%) of the PTB diagnosed had a cough of less than 2 weeks.

A TBscoreII ≥3 yielded the largest Area under the curve (AUC) for the HIV infected (0.62; 95% CI 0.53–0.72) while cough >2 weeks reached the largest AUC for the HIV uninfected (0.68; 95% CI 0.63–0.74) and the whole cohort (0.66; 95% CI 0.62–0.71). Self-reported weight loss had the lowest LR in the HIV infected (0.2). For the HIV uninfected and the whole cohort, a TBscore ≥3 resulted in the lowest LR (0.2 and 0.3, respectively). A TBscoreII ≥2 had a LR of 0.4 in the HIV uninfected and the whole cohort.

**Fig. 2:**
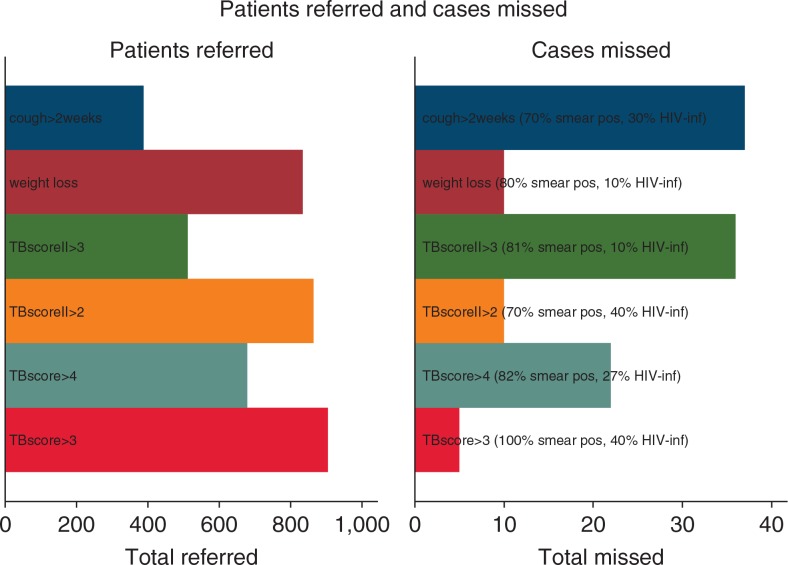
Referred patients and missed PTB cases using selected predictors as criterion.

Had we used the WHO applied criterion for TB suspicion (i.e. chronic cough; cough >2 weeks), almost one third (32.1%) of the sputum smear positive cases would have been missed. Among the other predictors, the one missing the least cases was a TBscore ≥3 (6.2%) (Fig. 2).

## Discussion

In this PhD thesis, it has been shown that TBscore has a better inter-observer reliability than one of the most used clinical rating scales in TB research, the KPS. However, the TBscore consisted of signs and symptoms with an unknown underlying correlation pattern and with partly high inter-observer variability, which decreased the overall reliability of the score. The proposed TBscoreII consists of related and reliable variables. Both TBscore and TBscoreII worked well in two quite different settings when used to predict failure and mortality. Finally, TBscore and TBscoreII were shown to be useful in case finding.

### TBscore versus KPS in TB

The widest applied rule to assess disease severity and predict outcome for TB patients is the KPS (22), which is why we chose to compare TBscore with it when scoring the same group of patients.

While both scores showed moderate agreement when used to score the same patient by two observers, the KPS ratings only fell into two of its three SC, indicating the inability to distinguish between patients moderately and seriously affected by PTB. This might be due to more disease-specific parameters used in TBscore. It has been postulated earlier that the KPS might not be useful other than in cancer patients (21, 67). The subjective assessment (i.e. the physician ranking the patient’s subjective experience of own illness) might obscure disease severity compared to the more objective and clinically based nature of the TBscore. This is also supported by the finding that HIV-status affected the KPS ratings; there were significantly more HIV-infected patients in the higher SC, which was not seen for the TBscore. Furthermore, when evaluating the scores prediction of unsuccessful outcome, TBscore showed a trend (insignificant) toward predicting treatment failure, death, or default, whereas KPS was unable to do so.

### Response of TBscore and TBscoreII to treatment effect and prediction of failure

The TBscore and TBscoreII worked well in both Ethiopia and Guinea-Bissau although they were slightly more responsive to treatment in Ethiopia. This might be due to the difference in baseline disease severity. The PTB patients from Ethiopia had a higher TBscore and TBscoreII at baseline than the Guinean patients, with the main contributors to higher scores being BMI and MUAC. It has been shown previously that malnutrition is more prevalent in Ethiopia than in West Africa (68), so one might expect higher scores in Ethiopia.

Failure to decrease TBscore/TBscoreII by ≥25% was associated with subsequent failure and mortality; though not always significant, the trend was seen in both settings and for both scores. Up to now, the most used predictors are sputum conversion and weight gain, as recommended by the WHO (45). It has been shown previously that sputum conversion has a low sensitivity to predict failure and the authors conclude that there is a low probability that a positive sputum smear at any month could correctly predict failure (47). Weight gain in TB patients during treatment is deceptive; the weight gained is mostly due to an increase in fat mass while the loss of muscle and organ tissue might be ongoing (52, 53). Further the measure is not well defined and in a previous study it could not predict outcome when measured at the end of the first month or the initial 2 months (69).

While this is the first external validation of TBscoreII, TBscore has been shown to predict poor outcome well in Ethiopian PTB patients (70).

### TBscore versus TBscoreII

Originally, TBscore consisted of five self-reported symptoms and six clinically assessed signs with varying reliability when assessed by two independent observers. The EFA done to uncover underlying constructs revealed that temperature >37°C, pulse, and hemoptysis were unrelated to the other items. The variables chosen for TBscoreII are reliable and related; hence, TBscoreII might be an improved outcome measure, though this was not as clear in Guinea-Bissau as in Ethiopia. Further, items requiring medical training (i.e. lung auscultation) and measures depending upon equipment not always available at basic health centers (thermometers and 30-second timers) are excluded in TBscoreII improving its overall applicability.

### Case finding using TBscore and TBscoreII

Currently applied indicators for possible PTB infection (cough >2 weeks for the HIV uninfected (45) and cough/weight loss/fever/night sweats for the HIV infected (71)) are insufficient in settings such as Bissau, where HIV status is often unknown at first encounter and sputum smear microscopy and CXR are the only available diagnostic tools.

Acknowledging this, the WHO recently changed its approach as to when to suspect TB (11), dismissing the previous focus on chronic cough. However, the current recommendations are vague and lack structured guidance for health-care workers in low-resource settings.

A CPR might help the overworked and under experienced nurse or physician to systematically sort out patients in need for further diagnostic measures. The diagnostic potential for all investigated tools was better than chance (i.e. the AUC was higher than 0.5) but none of them had an AUC above 0.75 which has been stated to be the threshold value for clinical usefulness (72). However, we hypothesized that some could hold predictive ability as to exclude PTB and found that absence of a TBscore ≥3 and self-reported weight loss declined the possibility of PTB by at least 25% though different in the HIV infected and the uninfected. The absence of a TBscore ≥2 declined the possibility for PTB by 20% in the HIV uninfected and the whole cohort. This indicates that screening with a clinical score consisting of easily assessable and reliable items might help sort out patients who do not need referral to further diagnostic tests, that is, an approach which might improve case finding while better diagnostic tools are still lacking. Whether TBscore or TBscoreII should be preferred is not clear from the present study and requires further research. Although the applicability is better for TBscoreII, it may have a lower predictive ability due to fewer included items and it does not seem to work as well in HIV-infected patients.

### Limitations

There is no capacity to carry out diagnostic sputum culture in Bissau or Gondar; hence, none of the SN PTB patients are culture confirmed. Nonetheless, all patients are diagnosed following WHO’s diagnostic guidelines (73), and followed through a diagnostic algorithm, which previously has been shown to have 89% sensitivity and 84% specificity toward PTB (74). While this reflects reality, it causes uncertainty in the evaluation of the diagnostic and predictive abilities of the investigated variables. It has been postulated that the increase in SN cases due to HIV could result in over-diagnosing of TB (75). This would dilute our samples and decrease the predictive and diagnostic ability of the investigated items.

The PTB patients from Gondar analyzed in paper II had a higher prevalence of sputum smear-positive PTB and HIV infection. Though a limitation, it could also be seen as strength, since TBscore and TBscoreII work well in both settings despite the differences.

Finally, it can be argued that there might be items overlooked in the initial variable-selection process. However, TBscore was developed following guidelines for score development (76) and the variables were chosen using the WHO clinical manuals list of important symptoms in TB (i.e. variables selected by a group of experts) (73) taking into account the caveats of using self-reported variables opposed to objectively measured ones. From the relevant variables, sputum production, loss of appetite, and presence of fatigue and clubbing were excluded; the former three due to missing collection of the data in the early part of the cohort and clubbing due to its rare presence (16). Including them may have improved the TBscore, but it could also have clouded its predictive ability. Among the originally chosen items, fever (77), low bodyweight (78, 79), and anemia (80) are well-known predictors of mortality in TB patients. Though well-known symptoms in TB patients, neither cough, hemoptysis, dyspnea, chest pain, night sweats nor findings at lung auscultation have been shown to predict mortality. Among the non-included items, only anorexia has been shown to associate with mortality (77).

## Conclusion and future perspectives

There is a void in the current approach of risk-grading PTB patients with regard to failure and mortality during treatment which could be filled by TBscore/TBscoreII. Thereby, the limited possibilities for a focused follow-up could be directed toward the ones most in need and limited resources could be used appropriately.

Further research is needed to elucidate if TBscore/TBscoreII has a general place in case finding. If our findings are repeated in other settings, TBscore/TBscoreII may become part of a future screening-routine, both passive and active, currently missing and thereby improving case finding.
